# Enhancing the Energy Storage Performance of Flexible Na_0.5_Bi_0.5_TiO_3_-Based Relaxor Thin Films Through a Relaxor Strategy

**DOI:** 10.3390/ma19153195

**Published:** 2026-07-27

**Authors:** Shibing Xiao, Huajun Sun, Huiting Sui

**Affiliations:** 1 College of Chemistry and Material Science, Hengyang Normal University, Hengyang 421008, China; sbxiao@hynu.edu.cn; 2 School of Materials Science and Engineering, Wuhan University of Technology, Wuhan 430070, China; 3 School of Physics and Optoelectronic Engineering, Ludong University, Yantai 264000, China

**Keywords:** relaxor ferroelectric, domain, flexible thin films, energy storage, Na_0.5_Bi_0.5_TiO_3_

## Abstract

Dielectric capacitors are employed in defense and automotive applications owing to their ultrahigh charge–discharge rates. To mitigate the high leakage current density of Na_0.5_Bi_0.5_TiO_3_ (NBT), SrTiO_3_ (STO), which exhibits excellent insulation performance, is incorporated into the NBT lattice to enhance both the breakdown field strength and the relaxor characteristics. Furthermore, the ionic radius of Sr^2+^ (0.1180 nm) is slightly larger than the average ionic radius of (NaBi)^2+^ (0.1025 nm). As a result, the introduction of STO induces lattice distortion, disrupts long-range ordering, and promotes the formation of short-range ordered domains, thereby strengthening the relaxor behavior (the relaxation degree γ increased from 1.63 to 1.84). Consequently, the 0.95(Na_0.5_Bi_0.5_)(Fe_0.02_Ti_0.99_)O_3_-0.05SrTiO_3_ thin film achieves a recoverable energy storage density (*W*_rec_) of 43.88 J/cm^3^ and an efficiency (*η*) of 73.95%. In addition, the thin film exhibits excellent temperature stability over a range of 10 to 170 °C, good frequency stability from 0.1 to 2.0 kHz, and robust fatigue endurance up to 1 × 10^8^ switching cycles. This work provides reliable technical and theoretical guidance for the application of NBT-based materials in energy storage.

## 1. Introduction

In recent years, with the advancement of electronic devices toward miniaturization and integration, dielectric thin-film capacitors, as an emerging high-speed energy storage technology, attracted widespread attention and held significant application prospects in fields such as defense, healthcare, and automotive [1,2,3]. Consequently, the dielectric energy storage film of this system exhibited a lower breakdown field strength, which hindered the achievement of excellent energy storage performance. Two key parameters characterize energy storage performance: energy storage density (*W*_rec_) and efficiency (*η*). These could be calculated using the following formulas [4,5,6,7,8].

(1) $$W_{\mathrm{rec}}=\int_{P_{r}}^{P_{m}}Edp$$

*η* = *W*_rec_/(*W*_rec_ + *W*_loss_) × 100% (2)

where *P*_m_ was the maximum polarization, *P*_r_ was the remanent polarization, and *W*_loss_ represented the energy dissipated during operation. Therefore, improving *W*_rec_ and *η* primarily relied on achieving both a large polarization difference (Δ*P* = *P*_m_ − *P*_r_) and a high breakdown field strength (*E*_BD_).

Relaxor ferroelectric materials possess fine ferroelectric domains, which can greatly reduce the cooperative coupling between ferroelectric domains. This gives the materials a high dielectric constant, high saturation polarization, and low remnant polarization, manifested as a slim hysteresis loop, and thus holds significant application potential in dielectric energy storage [9]. Na_0.5_Bi_0.5_TiO_3_ (NBT), as an environmentally friendly relaxor ferroelectric material, offered advantages such as high saturation polarization (*P*_s_~38 μC/cm^2^) and high Curie temperature (*T*_c_~320 °C) for energy storage applications [10,11]; however, its application was greatly hindered by issues including large leakage current density (up to 3.1 × 10^−3^ A/cm^2^) and low breakdown field strength [12,13]. In response to this challenge, the 0.94BaZr_0.2_Ti_0.8_O_3_-0.06Na_0.5_Bi_0.5_TiO_3_ thin film was prepared, achieving a *W*_rec_ of 62.08 J/cm^3^ and *η* of 85.18% under an ultrahigh breakdown field strength (*E*_BD_) of 6117 kV/cm [14]. Besides, Cheng obtained high *P*_m_ (112.7 µC/cm) and low *P*_r_ (14.9 µC/cm) by optimizing polarization response, ultimately achieving excellent energy storage properties with *W*_rec_ ≈ 81.9 J/cm^3^ and *η* ≈ 64.4% at an electric field of 2285 kV/cm in a flexible Mn-doped 0.97(0.93Na_0.5_Bi_0.5_TiO_3_-0.07BaTiO_3_)-0.03BiFeO_3_ inorganic thin-film capacitor, with the assistance of a two-dimensional fluorophlogopite mica substrate. This was mainly attributed to the high degree of relaxor dispersion in the film, as well as the coexistence of ferroelectric and antiferroelectric phases [15]. In addition, Lee and Son incorporated BaTiO_3_ into the lattice of NBT to optimize its relaxation properties, reducing the residual polarization of NBT from 28.4 to 4.7 µC/cm, ultimately achieving a *W*_rec_ of 54.3 J/cm^3^ and *η* of 89.5% in 0.94(Na_0.5_Bi_0.5_TiO_3_-0.06BaTiO_3_ thin film [16]. In addition to relaxor ferroelectrics, the high breakdown field strength and low *P*_r_ exhibited by paraelectric dielectrics in energy storage applications have also attracted considerable attention. For example, Peng et al. prepared Ba_0.7_Sr_0.3_Fe_x_Ti_1−x_O_3_ paraelectric thin film capacitors using the sol-gel method, achieving a *W*_rec_ of only 7.6 J/cm^3^ and an *η* of only 65% [17]. Furthermore, they introduced Al_2_O_3_ nanoparticles into the SrTiO_3_ thin film. The Al_2_O_3_ nanoparticles created ion transport channels, which promoted an anodic oxidation reaction under an applied electric field, forming an Al_2_O_3_ − δ layer at the Al/SrTiO_3_ interface. As a result, the breakdown field strength increased from 2330 kV/cm to 5070 kV/cm, and *W*_rec_ increased from 7.6 J/cm^3^ to 19.3 J/cm^3^. Moreover, guided by theoretical and phase-field simulations, remarkably superior comprehensive properties—including an ultrahigh *η* of 90~94% and a high *W*_rec_ of 85~90 J/cm^3^—were obtained in SrTiO_3_ by manipulating local symmetry breaking via the introduction of Ti/O defects [18]. However, due to the ultra-low dielectric constant or polarization strength of paraelectric dielectrics, their *W*_rec_ remained relatively low, which significantly limited their development and application. Thus, integrating the high polarization strength of a relaxor ferroelectric phase (NBT, high saturation polarization, and Curie temperature) with the high breakdown field strength of a paraelectric phase (SrTiO_3_, high *E*_BD_ but low *P*_m_) offered a promising research direction for optimizing the energy storage performance of dielectric thin films.

In this work, SrTiO_3_ was incorporated into the (Na_0.5_Bi_0.5_)TiO_3_ lattice to fabricate (1 − x)(Na_0.5_Bi_0.5_)(Fe_0.02_Ti_0.98_)O_3−x_SrTiO_3_ (x = 0.00, 0.05, 0.10, 0.15) thin films. The effects of SrTiO_3_ incorporation on domain response and relaxation behavior, as well as the underlying mechanism governing the energy storage performance, were systematically investigated. This study aims to enhance the *E*_BD_ and *P*_m_ of NBT-based thin-film capacitors, thereby improving their energy storage capability, and simultaneously provides both theoretical and experimental guidance for energy storage applications in the NBT system.

## 2. Experimental

The (1 − x)(Na_0.5_Bi_0.5_)(Fe_0.02_Ti_0.98_)O_3−x_SrTiO_3_ (x = 0.00, 0.05, 0.10, 0.15) thin films, designated as NBS_0_FT, NBS_5_FT, NBS_10_FT, and NBS_15_FT, respectively, are fabricated via the sol–gel method. The precursor solutions are prepared using raw materials including titanium (IV) butoxide [C_4_H_9_O)_4_Ti] (Shanghai Aladdin Biochemical Technology Co., Ltd., Shanghai, China), bismuth nitrate pentahydrate (Bi(NO_3_)·5H_2_O) (Sinopharm Chemical Reagent Co., Ltd., Shanghai, China), iron (III) nitrate nonahydrate (Fe(NO_3_)_3_·9H_2_O) (Sinopharm Chemical Reagent Co., Ltd., Shanghai, China), strontium acetate hemihydrate ((CH_3_COO)_2_Sr·½H_2_O) (Shanghai Macklin Biochemical Co., Ltd., Shanghai, China), and sodium acetate (CH_3_COONa) (Sinopharm Chemical Reagent Co., Ltd., Shanghai, China). To stabilize the solution, C_4_H_9_O)_4_Ti is dissolved in acetylacetone (Sinopharm Chemical Reagent Co., Ltd., Shanghai, China). A mixture of ethanol and ethylene glycol (Sinopharm Chemical Reagent Co., Ltd., Shanghai, China) is used to dissolve (Bi(NO_3_)·5H_2_O), (Fe(NO_3_)_3_·9H_2_O), and CH_3_COONa, while ((CH_3_COO)_2_Sr·½H_2_O) is dissolved in acetic acid (Tianjin Hengxing Chemical Reagent Manufacturing Co., Ltd., Tianjin, China). Each solution is magnetically stirred for 12 h, then combined and stirred for an additional 24 h. High-quality precursor solutions are obtained after allowing the mixture to stand for 48 h.

Flexible Pt/Mica substrates are employed to grow the thin films. The precursor solution is spin-coated at 3000 rpm for 30 s, dried at 200 °C for 3 min, and annealed at 600 °C for 15 min. This coating–drying–annealing cycle is repeated to achieve the desired film thickness. Gold top electrodes with a diameter of 0.4 mm are deposited onto the films through a shadow mask. The crystallinity of the films is analyzed by X-ray diffraction (XRD, D8, Bruker, Billerica, MA, USA). Morphology is examined using field-emission scanning electron microscopy (FESEM, S-4800, Hitachi, Tokyo, Japan) and atomic force microscopy (AFM, Dimension Edge, Santa Barbara, CA, USA). Domain structures are characterized via piezoelectric force microscopy (PFM, Dimension Edge, Santa Barbara, CA, USA). Ferroelectric and dielectric properties are evaluated using a ferroelectric tester (Radiant, RTI-Multiferroic, Albuquerque, NM, USA) and a dielectric analyzer (Concept 80, Novocontrol, Montabaur, Germany), respectively.

## 3. Results and Discussions

**Figure 1:**
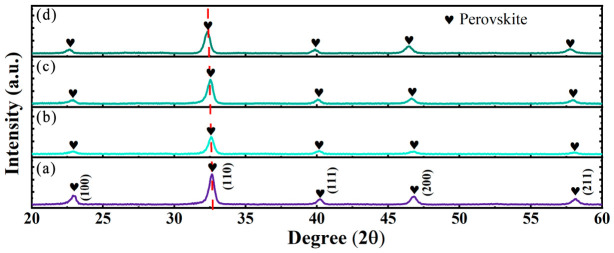
The GIXRD patterns of (1 − x)(Na_0.5_Bi_0.5_)(Fe_0.02_Ti_0.98_)O_3−x_SrTiO_3_ thin films: (**a**) x = 0.00, (**b**) x = 0.05, (**c**) x = 0.10, and (**d**) x = 0.15.

Figure 1 shows the grazing incidence X-ray diffraction (GIXRD) patterns of thin films with varying amounts of SrTiO_3_ solid solution in (Na_0.5_Bi_0.5_)(Fe_0.02_Ti_0.99_)O_3_. As can be seen from the figure, all (1 − x)(Na_0.5_Bi_0.5_)(Fe_0.02_Ti_0.99_)O_3−x_SrTiO_3_ flexible thin films exhibit a pure polycrystalline perovskite phase (Ref. ICSD #154040 of NBT phases), with no other impurity phases detected within the measurable range of the equipment. Additionally, as SrTiO_3_ is introduced into the (Na_0.5_Bi_0.5_)(Fe_0.02_Ti_0.99_)O_3_ lattice, the (110) peak shifts toward lower angles. This is due to the larger ionic radius of Sr^2+^ (r_1_ = 0.1180 nm) compared to the average ionic radius of (NaBi)^2+^ (r_2_ = 0.1025 nm). Therefore, when Sr^2+^ is incorporated into the (Na_0.5_Bi_0.5_)(Fe_0.02_Ti_0.99_)O_3_ lattice, it causes lattice expansion. According to Bragg’s law (2dsinθ=nλ, *d* is the distance between adjacent crystal planes, *λ* is the wavelength of the X-ray, *θ* is the angle between the X-ray and the crystal plane, and n is the diffraction order) [19], an increase in the d-spacing results in a decrease in the *θ* value, which manifests as a peak shift toward lower angles in the XRD pattern. This also confirms the successful incorporation of Sr^2+^ into the (Na_0.5_Bi_0.5_)(Fe_0.02_Ti_0.99_)O_3_ lattice.

**Figure 2:**
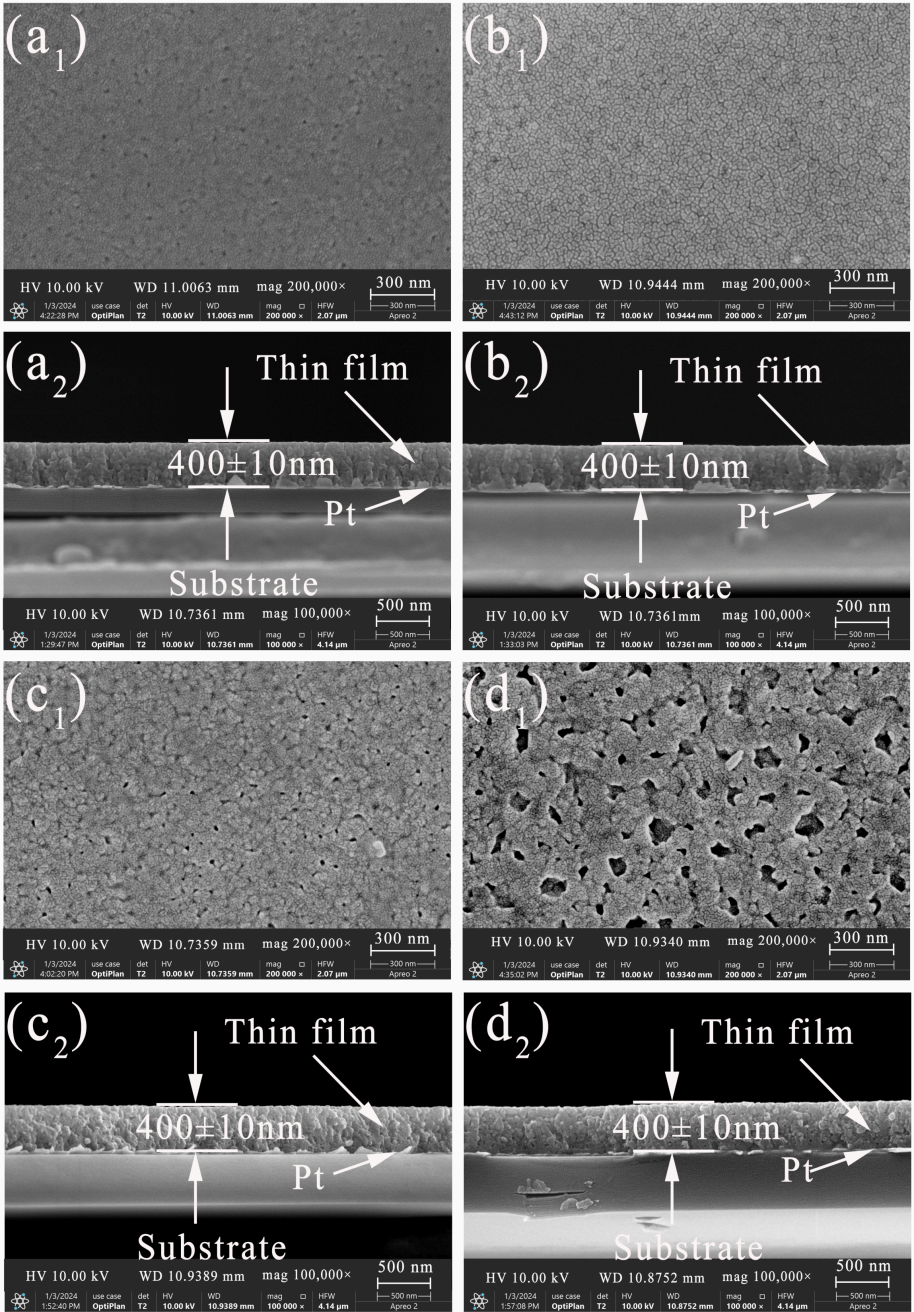
The SEM images of (1 − x)(Na_0.5_Bi_0.5_)(Fe_0.02_Ti_0.98_)O_3−x_SrTiO_3_ thin films: (**a_1_**,**a_2_**) NBS_0_FT, (**b_1_**,**b_2_**) NBS_5_FT, (**c_1_**,**c_2_**) NBS_10_FT and (**d_1_**,**d_2_**) NBS_15_FT.

Figure 2 shows the surface and cross-sectional SEM morphologies of the (1 − x)(Na_0.5_Bi_0.5_)(Fe_0.02_Ti_0.99_)O_3_ thin films. When x = 0.00, the NBS_0_FT film surface exhibits a small number of pores and voids, which result from the volatilization or carbonization of organic compounds during the film preparation process [20], as shown in Figure 2(a_1_). When x = 0.05, after SrTiO_3_ is introduced into the (Na_0.5_Bi_0.5_)(Fe_0.02_Ti_0.99_)O_3_ lattice, the pores on the film surface almost disappear, and the structure becomes dense, indicating that an appropriate amount of SrTiO_3_ can promote grain growth, as shown in Figure 2(b_1_). A dense structure can enhance the electrical insulation properties of the film, increase the breakdown field strength, and thereby improve the energy storage performance. However, as the SrTiO_3_ content increases, pores and voids reappear on the surface. When the SrTiO_3_ doping amount reaches 15%, the surface pores and voids significantly increase, as shown in Figure 2(c_1_,d_1_). This is because excess Sr^2+^ ions are not fully incorporated into the (Na_0.5_Bi_0.5_)(Fe_0.02_Ti_0.99_)O_3_ lattice. These excess Sr^2+^ ions may accumulate at grain boundaries or interstitial sites, acting as defects that impede nucleation and growth of grains. Additionally, Figure 2(a_2_,b_2_,c_2_,d_2_) present the cross-sectional SEM images of the corresponding films. It can be observed that all films exhibit no obvious pores or voids in their cross-sections, indicating a dense internal structure. Furthermore, the thickness of all film samples is approximately 400 nm, and the similar thickness helps avoid errors in results and analysis due to size effects.

**Figure 3:**
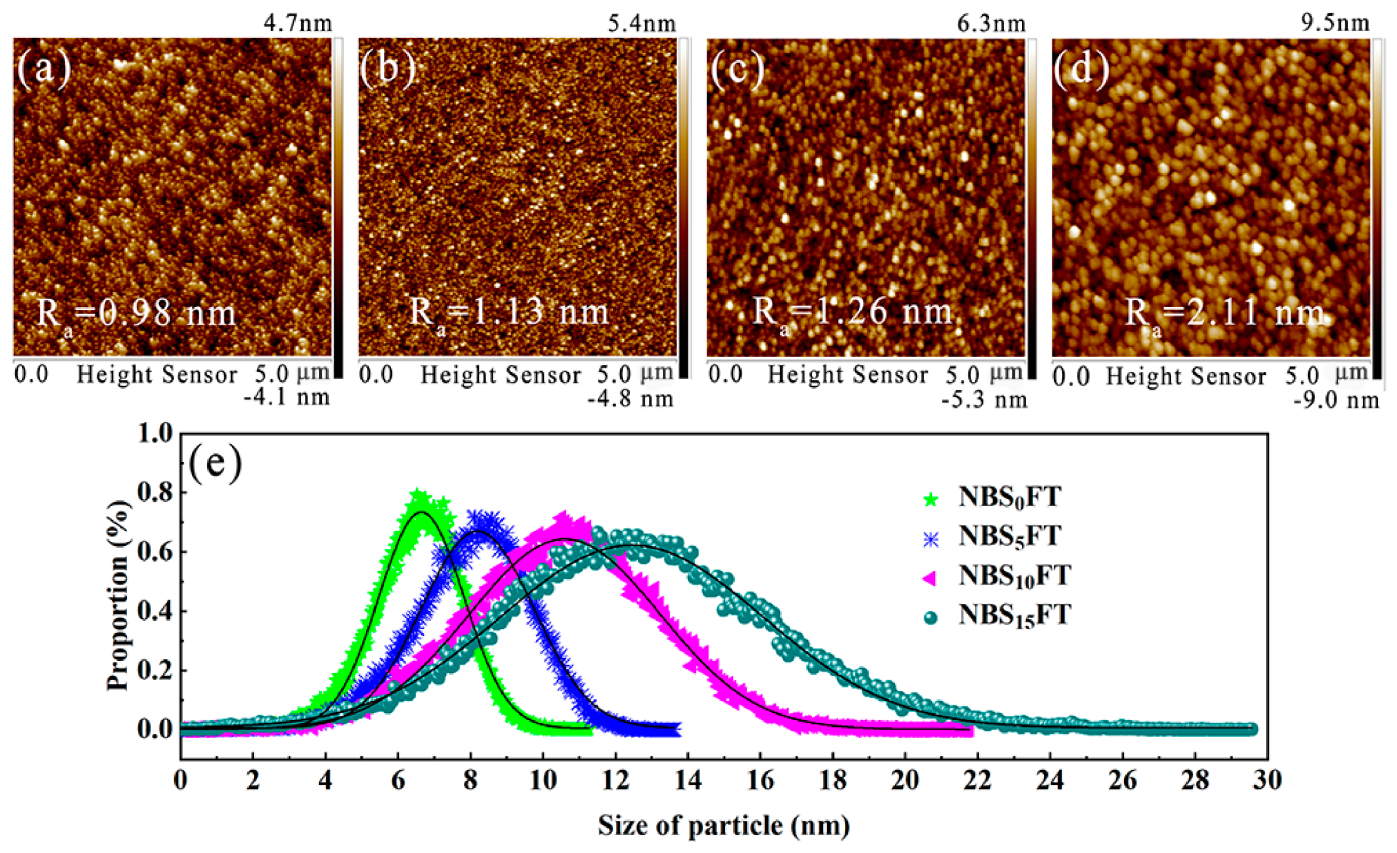
The AFM images of (1 − x)(Na_0.5_Bi_0.5_)(Fe_0.02_Ti_0.98_)O_3−x_SrTiO_3_ thin films: (**a**) NBS_0_FT, (**b**) NBS_5_FT, (**c**) NBS_10_FT and (**d**) NBS_15_FT. (**e**) The corresponding distribution of particles.

To further analyze the surface morphology and grain growth of the films, a 5 × 5 μm micro-area is scanned using AFM, as shown in Figure 3. From Figure 3a–d, it can be observed that the NBS_5_FT film surface has fewer pores, while the other films exhibit pores and voids, which is consistent with the SEM results. The root mean square roughness (*R*_a_) values for these films are 0.98 nm, 1.13 nm, 1.26 nm, and 2.11 nm, respectively. The *R*_a_ values of the first three samples are relatively close, while the roughness of the last sample shows a significant increase. The variation in roughness is closely related to grain size, morphology, and the presence of pores. Higher surface roughness can lead to the expansion of leakage current pathways, which in turn increases leakage current [21]. Figure 3e shows the statistical distribution of surface grains for all film samples, all of which follow a normal distribution, indicating uniform grain size distribution. The grains of the NBS_0_FT and NBS_5_FT films are significantly finer and more concentrated compared to the other two samples, suggesting that these films have smaller and more uniformly distributed grains. This trend is consistent with the *R*_a_ values.

**Figure 4:**
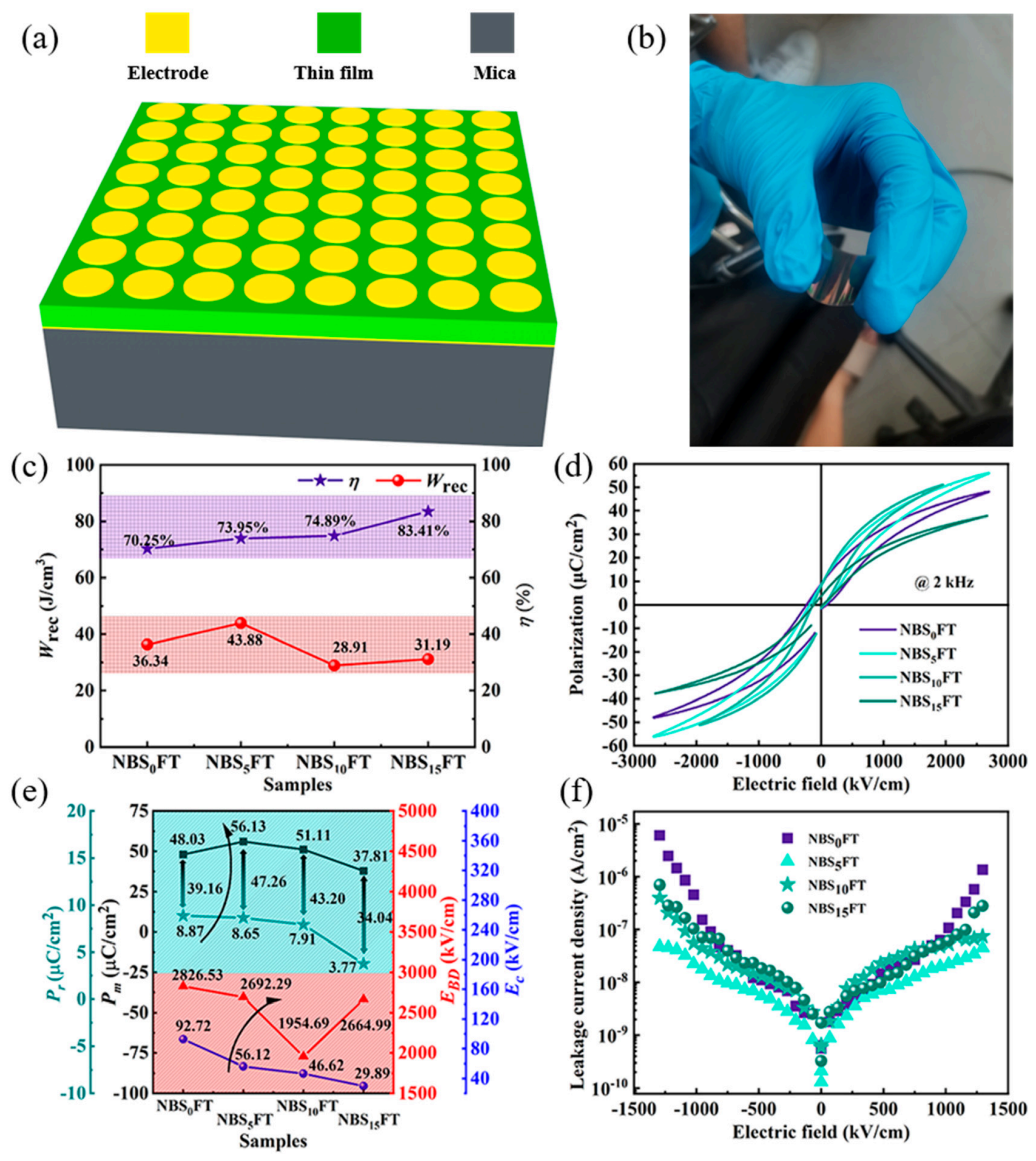
(1 − x)(Na_0.5_Bi_0.5_)(Fe_0.02_Ti_0.98_)O_3−x_SrTiO_3_ thin films for: (**a**) Schematic diagram, (**b**) Photograph, (**c**) *W_rec_* and *η,* (**d**) *P*-*E* loops, (**e**) *P*_m_, *P*_r_, *P*_m_ − *P*_r_, *E*_BD_ values, and *E*_c,_ and (**f**) *I*/*E* curves.

Figure 4a shows the *W*_rec_ and *η* of all (1 − x)(Na_0.5_Bi_0.5_)(Fe_0.02_Ti_0.98_)O_3−x_SrTiO_3_ flexible thin-film capacitors. As can be seen, the *η* of all flexible thin-film capacitors is above 70% and increases with the SrTiO_3_ solid solution content. Meanwhile, *W*_rec_ shows a trend of first increasing and then decreasing, with the NBS_5_FT film achieving the highest *W*_rec_ of 43.88 μC/cm^3^. Simultaneously, the *η* of this film also reaches 73.95%. This excellent energy storage performance is primarily related to its high *E*_BD_ and (*P*_m_ − *P*_r_) values. Figure 4b presents the corresponding hysteresis loops, all of which exhibit typical “slim” and “waist-pinched” hysteresis loops. Such hysteresis loops enable both high *W*_rec_ and *η*. To more intuitively demonstrate the performance of these flexible thin-film capacitors, their respective *P*_m_, *P*_r_, (*P*_m_ − *P*_r_), *E*_BD_, and *E*_c_ values are displayed in Figure 4c. As shown, with the introduction of SrTiO_3_ into the (Na_0.5_Bi_0.5_)(Fe_0.02_Ti_0.98_)O_3_ lattice, *P*_m_ first increases and then decreases, while *P*_r_ shows a continuous decreasing trend, resulting in (*P*_m_ − *P*_r_) also first increasing and then decreasing. This indicates that an appropriate amount of SrTiO_3_ solid solution (i.e., 5% wt.) can enhance the polarization intensity of (Na_0.5_Bi_0.5_)(Fe_0.02_Ti_0.98_)O_3_ thin-film capacitors. This is because an appropriate amount of Sr^2+^ doping can disrupt the long-range ordered domains in the (Na_0.5_Bi_0.5_)(Fe_0.02_Ti_0.98_)O_3_ film, forming fine ferroelectric domains, which is widely reported in previous studies [22,23,24,25,26]. Under an applied electric field, the reduced hindrance to reorientation allows fine ferroelectric domains to align at lower fields, yielding a higher maximum polarization (*P*_m_) and a lower coercive field. Upon field removal, the fine ferroelectric domains readily return to their initial randomly oriented state with low energy, resulting in a low remnant polarization (*P*_r_). The presence of fine ferroelectric domains thus increases *P*_m_ and decreases *P*_r_, enhancing the recoverable energy density (*W*_rec_) and efficiency (*η*) of flexible thin-film capacitors. However, excessive Sr^2+^ incorporation leads to the formation of a substantial amount of paraelectric SrTiO_3_ [27], which increases the leakage current and reduces the breakdown strength, thereby deteriorating the energy storage density and efficiency. One of the typical characteristics of paraelectric dielectrics is their low polarization intensity, which also explains the reduction in *P*_m_ and *P*_r_ values in NBS_10_FT and NBS_15_FT films. The decrease in *P*_r_ is the main reason for the monotonic increase in *η*. Additionally, the *E*_BD_ of the flexible ferroelectric thin film capacitors first increases and then decreases, reaching its maximum value in the NBS_5_FT film capacitor. This is because the NBS_5_FT film has a smooth surface morphology, uniformly distributed grains, and a dense microstructure. However, when the Sr^2+^ doping amount reaches 15%, the *E*_BD_ increases again, which is attributed to the presence of a large amount of high-breakdown-performance SrTiO_3_ in the film. The improvement in *E*_BD_ is the main reason for the enhanced *W*_rec_ in the NBS_5_FT film capacitor.

Leakage current density is one of the important parameters characterizing ferroelectric thin films and can serve as a key indicator for assessing the quality of ferroelectric films. The relationship between leakage current density and electric field for different thin-film capacitors is shown in Figure 4d. As can be seen, the introduction of a certain amount of SrTiO_3_ into the (Na_0.5_Bi_0.5_)(Fe_0.02_Ti_0.98_)O_3_ lattice reduces the leakage current density from the order of 10^−5^ A/cm^2^ to 10^−7^ A/cm^2^. The NBS_5_FT film capacitor exhibits the lowest leakage current density, which is closely related to its smooth surface morphology, uniformly distributed grains, and dense microstructure. In contrast, the leakage current density of NBS_10_FT and NBS_15_FT films increases, which is associated with the presence of numerous pores in these films.

**Figure 5:**
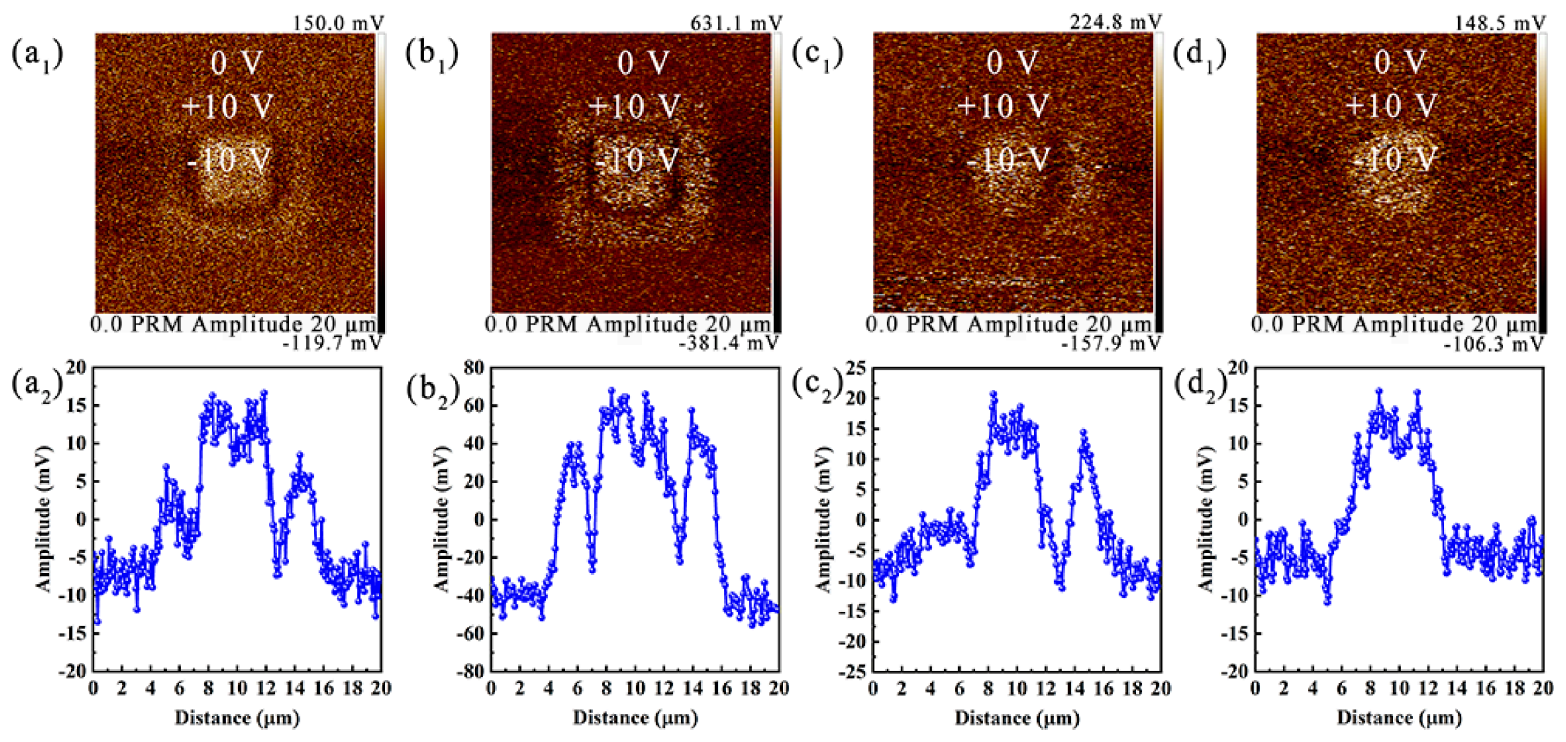
The amplitude of domain for (1 − x)(Na_0.5_Bi_0.5_)(Fe_0.02_Ti_0.98_)O_3−x_SrTiO_3_ flexible thin films after polarization with DC voltage of ±10 V and 0 V: (**a_1_**,**a_2_**) NBS_0_FT, (**b_1_**,**b_2_**) NBS_5_FT, (**c_1_**,**c_2_**) NBS_10_FT, and (**d_1_**,**d_2_**) NBS_15_FT.

To further investigate the polarization process of ferroelectric domains in the thin films, the amplitudes of the ferroelectric domains of several samples are measured using PFM. As shown in Figure 5(a_1_–d_1_), the four samples exhibit clear contrast between polarized and unpolarized regions, indicating the presence of ferroelectric domains in all samples. Additionally, ferroelectric domains align orderly under DC voltage excitation, while they vibrate under AC voltage. Under the same AC voltage, a larger vibration amplitude of the ferroelectric domains indicates a more significant response to the external electric field, suggesting higher polarization intensity. The NBS_5_FT and NBS_10_FT films display distinct “frame-like” patterns, with the NBS_5_FT sample being particularly evident, while the other two samples show weaker electric field responses from their ferroelectric domains. To quantitatively describe the electric field response of these ferroelectric domains, the amplitude values of each sample are extracted using Nanoscope analysis software (Version 1.40) and plotted in Figure 5(a_2_–d_2_). Comparison reveals that the response amplitude of the ferroelectric domains in the NBS_5_FT sample reaches 70 mV; the response amplitude of the ferroelectric domains in the NBS_10_FT film is only about 20 mV, while the response amplitudes of the ferroelectric domains in the other two samples are even lower, around 15 mV. These results are consistent with the analysis in Figure 4.

**Figure 6:**
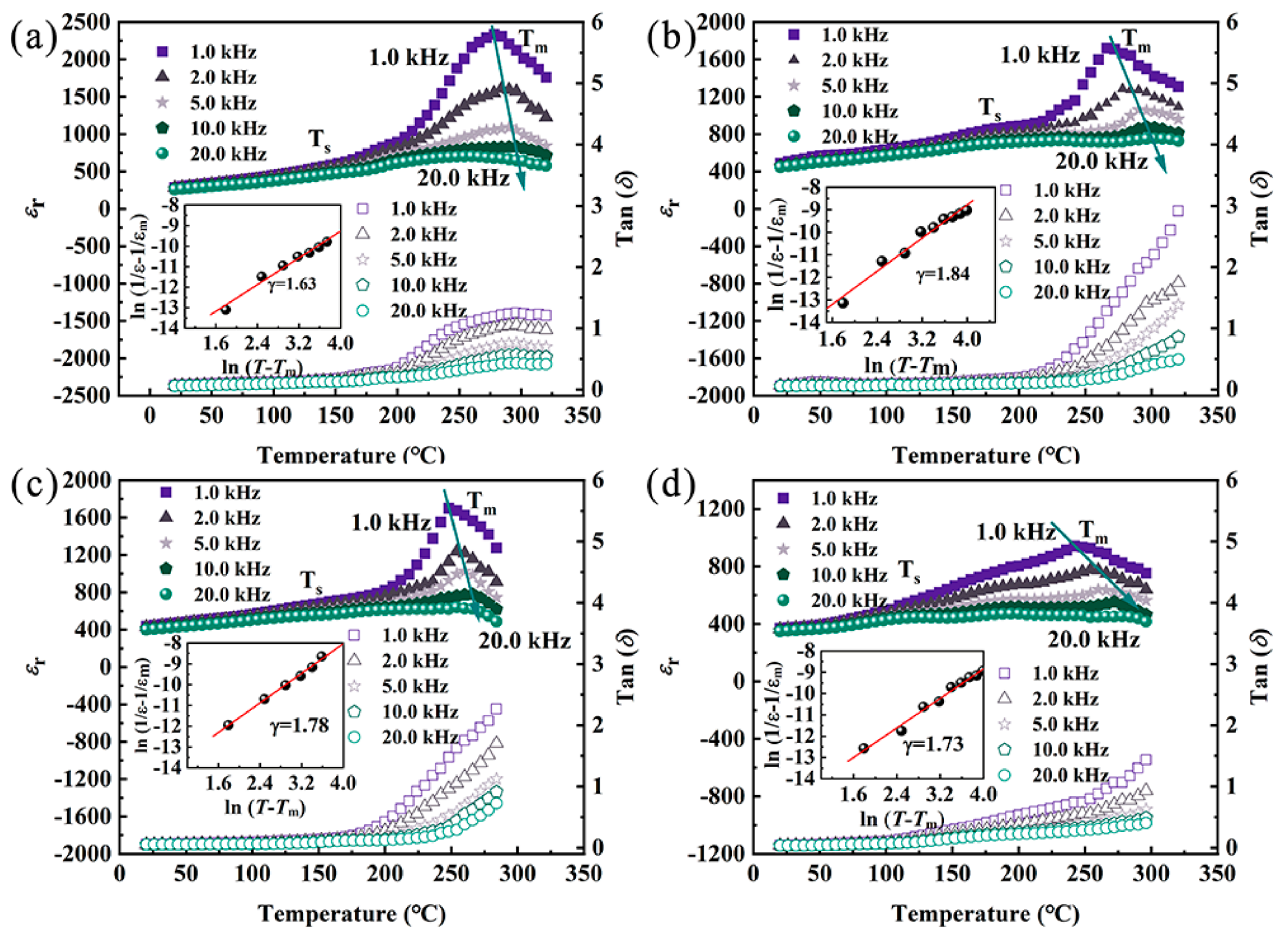
The temperature dependence of relative dielectric constant for (1 − x)(Na_0.5_Bi_0.5_)(Fe_0.02_Ti_0.98_)O_3−x_SrTiO_3_ thin films: (**a**) NBS_0_FT, (**b**) NBS_5_FT, (**c**) NBS_10_FT, and (**d**) NBS_15_FT. Inset give the corresponding ln(1/*ε* − 1/*ε*_m_) vs. ln(*T* − *T*_m_) at the frequency of 1.0 kHz for thin films.

Figure 6 shows the relationship between the dielectric constant and loss of all flexible thin films as a function of temperature. As observed, all films exhibit frequency dispersion in the range of 1~20.0 kHz, and the temperature corresponding to the maximum dielectric constant (*T*_m_) shifts toward higher temperatures, indicating that these films are relaxor ferroelectrics. Additionally, with the introduction of SrTiO_3_, the Tm values for the NBS_0_FT, NBS_5_FT, NBS_10_FT, and NBS_15_FT films are 278 °C, 266 °C, 248 °C, and 242 °C, respectively. Their *T*_m_ values are all above 240 °C, indicating good temperature stability of these relaxor ferroelectric thin films. Moreover, the Tm values show a decreasing trend, which is attributed to the reduction of Fe in the system as the SrTiO_3_ content increases, leading to a decrease in the BiFeO_3_ content in the system. The Curie temperature of pure BiFeO_3_ is as high as 850 °C [28]; therefore, when the BiFeO_3_ component in the system decreases, the Curie temperature of the entire system also decreases. The inset shows the relationship between ln(1/*ε* − 1/*ε*m) and ln(*T* − *T*_m_) for the corresponding films at 1.0 kHz, fitted according to the modified Curie-Weiss law ($1/\varepsilon_{r}-1/\varepsilon_{m}={\left(T-T_{m}\right)}^{\gamma }/C$) [22,29,30]. Clearly, the γ values for the flexible ferroelectric thin film capacitors are 1.63, 1.84, 1.78, and 1.73, respectively. All γ values are greater than 1.6, indicating good relaxor characteristics, and they show a trend of first increasing and then decreasing. Notably, the γ values of the films after SrTiO_3_ solid solution are all greater than 1.7, indicating that the introduction of Sr^2+^ effectively enhances the relaxor properties of the films. Among them, the NBS_5_FT film capacitor has a γ value of 1.84, indicating the best relaxor characteristics. This is because an appropriate amount of Sr^2+^ introduced into the lattice disrupts the long-range order of the lattice, forming a large number of fine ferroelectric domains.

**Figure 7:**
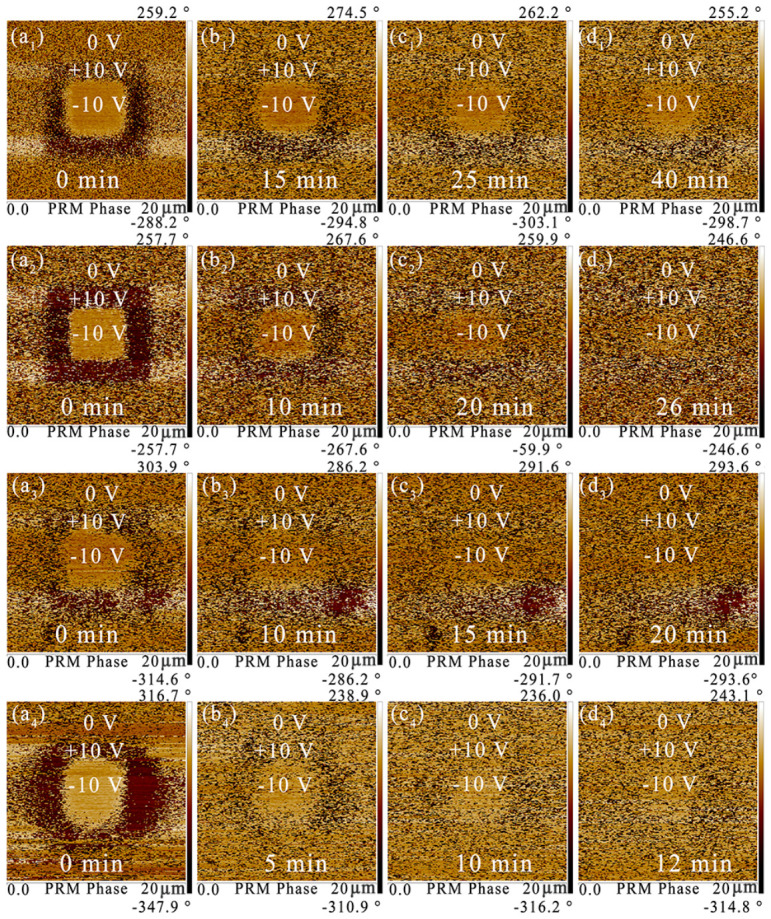
The phase of domain for (1 − x)(Na_0.5_Bi_0.5_)(Fe_0.02_Ti_0.98_)O_3−x_SrTiO_3_ flexible thin films under DC voltage of ±10 V and 0 V: (**a_1_**–**d_1_**) NBS_0_FT, (**a_2_**–**d_2_**) NBS_5_FT, (**a_3_**–**d_3_**) NBS_10_FT, and (**a_4_**–**d_4_**) NBS_15_FT.

To further study the relaxor properties of the flexible thin films, the phase evolution of the ferroelectric domains in each sample is tested. After removing the DC voltage, the same area is cyclically tested with an AC voltage amplitude of 6 V, obtaining phase diagrams of the ferroelectric domains at different times, as shown in Figure 7. Except for NBS_10_FT, the phase diagrams of the ferroelectric domains in the other samples show relatively clear “frame-like” patterns, indicating low energy required for ferroelectric domain switching during polarization, as shown in Figure 7(a_1_–a_4_). Among them, the NBS_5_FT film exhibits clear contrast, indicating that under the same polarization electric field, the domain wall movement in this film encounters less hindrance, and the ferroelectric domains switch more easily, corresponding to a higher polarization intensity. For NBS_10_FT, a large number of ferroelectric domains do not switch, which may be due to domain wall pinning on one hand, and on the other hand, the switched ferroelectric domains may return to their initial state before testing. After the DC electric field is removed, the depolarization process begins, and most ferroelectric domains gradually return to their initial state. The depolarization times for the NBS_0_FT, NBS_5_FT, NBS_10_FT, and NBS_15_FT films are 40 min, 26 min, 20 min, and 12 min, respectively, showing a gradually shortening trend. With the introduction of SrTiO_3_, the depolarization process of the films is effectively shortened, and the relaxor properties are enhanced, which is consistent with the results in Figure 6. However, although NBS_5_FT has stronger relaxor properties, its depolarization time is longer than that of NBS_10_FT and NBS_15_FT; this is because the increase in SrTiO_3_ content effectively reduces the *P*_r_ value of the material.

The NBS_5_FT flexible thin film capacitor exhibits excellent relaxor properties and achieves superior energy storage performance. However, in practical applications, the impact of different application environments on the energy storage performance of this thin film capacitor must be considered. Evaluating its stability and reliability is an essential step.

**Figure 8:**
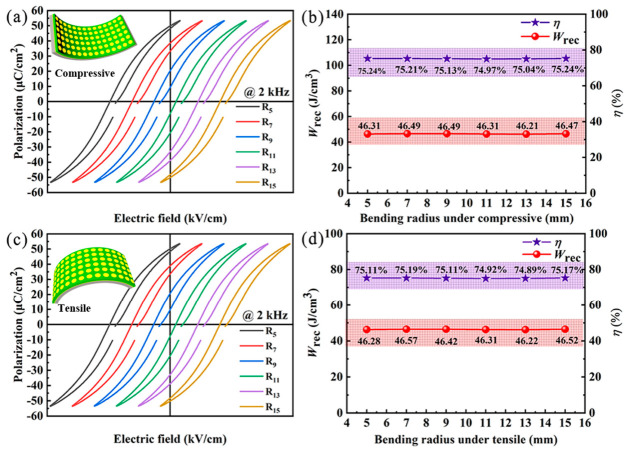
The energy storage performances of NBS_5_FT flexible thin film capacitor under tensile and compressive states with different bending radii: (**a**,**c**) *P-E* loops and (**b**,**d**) *W_rec_* and *η* values.

First, the hysteresis loops of the flexible NBS_5_FT thin film capacitor under different tensile and compressive radii, along with their corresponding *W*_rec_ and *η*, are shown in Figure 8. As observed, under tensile or compressive radii of 5 mm (R_5_), 7 mm (R_7_), 9 mm (R_9_), 11 mm (R_11_), 13 mm (R_13_), and 15 mm (R_15_), the hysteresis loops remain essentially unchanged; as a result, the *W*_rec_ and *η* of the thin film capacitor under different tensile and compressive radii are maintained at around 46 J/cm^3^ and 75%, respectively. The results indicate that this flexible thin film capacitor can leverage its good flexibility for application in specific environments involving stretching or compression, such as certain robotic arm joints.

**Figure 9:**
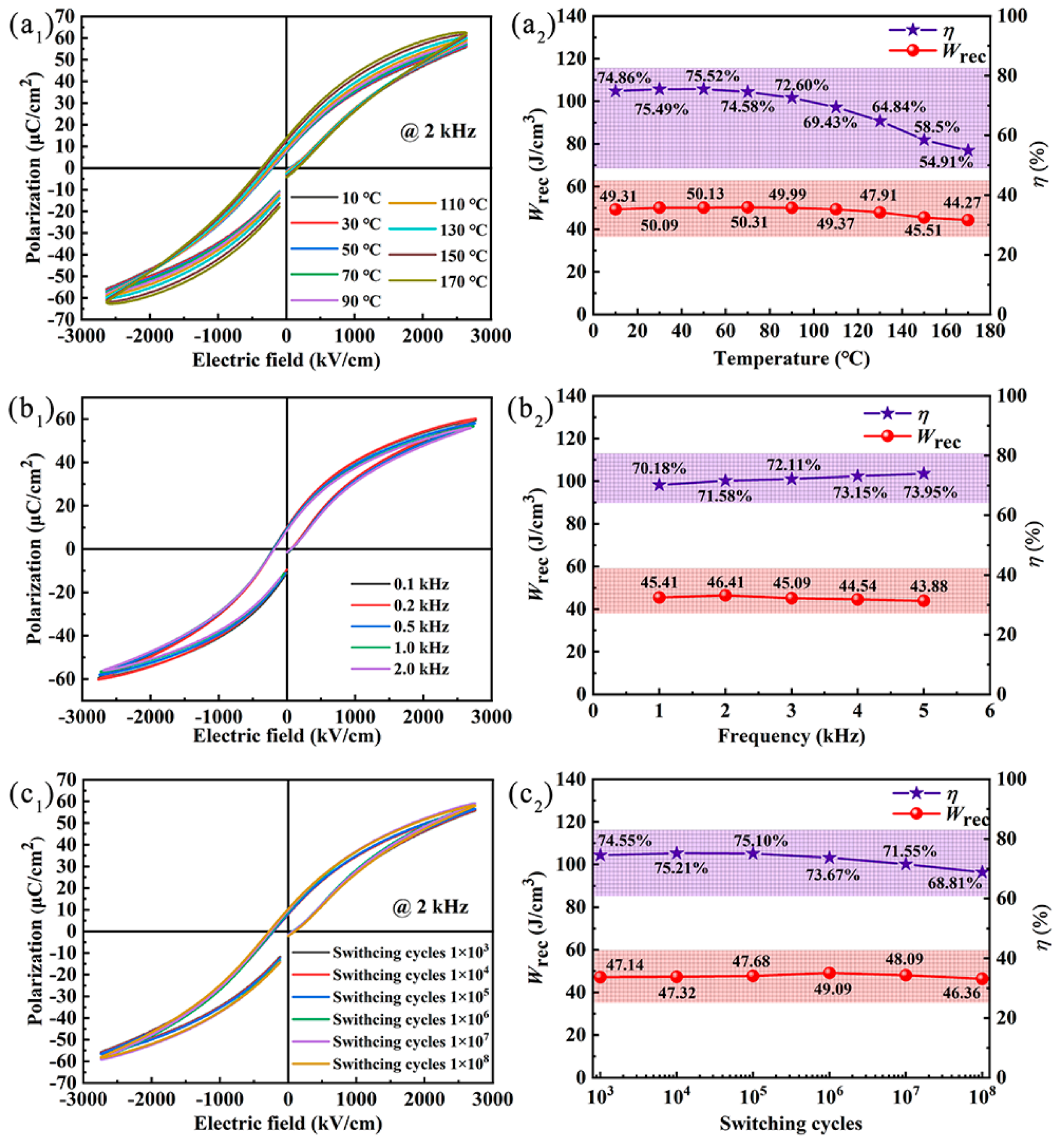
*P*-*E* loops and their corresponding *W_rec_* and *η* of NBS_5_FT thin film under different conditions: (**a_1_**,**a_2_**) temperatures, (**b_1_**,**b_2_**) frequencies, and (**c_1_**,**c_2_**) switching cycles.

In addition to bending resistance, temperature stability, frequency stability, and good fatigue resistance are also important indicators for evaluating energy storage thin films. Figure 9(a_1_,a_2_) show the hysteresis loops of the NBS_5_FT thin film capacitor at a frequency of 2.0 kHz and the corresponding *W*_rec_ and *η* as a function of temperature. In the temperature range of 10 °C to 170 °C, the hysteresis loops gradually deform as the temperature increases, as shown in Figure 9(a_1_). The *W*_rec_ and *η* of the NBS_5_FT film show an initial improvement, which is due to the presence of fine ferroelectric domains, whose activity increases with rising temperature. As the temperature continues to rise, the leakage current of the system increases, leading to the degradation of *W*_rec_ and *η*, as shown in Figure 9(a_2_,b_1_,b_2_), which displays the hysteresis loops at different frequencies (0.1 kHz to 2.0 kHz) and their corresponding *W*_rec_ and *η*. As the frequency increases, the *W*_rec_ remains around 45 J/cm^3^, while *η* increases from 70% to 74%, indicating that this thin film capacitor can be used at different frequencies. Fatigue resistance is another important application indicator for thin-film capacitors, characterizing the number of repeated switching cycles of ferroelectric domains. After 1 × 10^8^ charge-discharge cycles, the shape of the hysteresis loop of this thin film capacitor remains essentially unchanged, as shown in Figure 9(c_1_). The corresponding *W*_rec_ remains stable above 46 J/cm^3^, while *η* decreases from 74.55% to 68.81%, a reduction of only 6%, as shown in Figure 9(c_2_). It can be concluded that the NBS_5_FT thin film capacitor exhibits good fatigue resistance.

## 4. Conclusions

The ferroelectric domain structure of the thin films is modulated by adjusting the SrTiO_3_ content in the thin films. When the doping level is 5%, the 0.95(Na_0.5_Bi_0.5_)(Fe_0.02_Ti_0.98_)O_3_-0.05SrTiO_3_ film achieves a *W*_rec_ of 43.88 J/cm^3^ and an *η* of 73.95% under an electric field strength of 2692.29 kV/cm. Additionally, the NBS_5_FT film exhibits outstanding temperature stability (10~170 °C), excellent frequency stability (0.1~2.0 kHz), and robust fatigue resistance (1 × 10^8^ charge-discharge cycles). Meanwhile, it also has excellent bendability performance (bending radius: 5~15 mm). This work promotes the theoretical and applied development of dielectric energy storage materials.

## Data Availability

The original contributions presented in this study are included in the article. Further inquiries can be directed to the corresponding authors.
